# Research on clinical characteristics and prognostic analysis of heparin-induced thrombocytopenia after surgery for acute type a aortic dissection

**DOI:** 10.1186/s13019-021-01482-2

**Published:** 2021-04-20

**Authors:** Chu-zhi Zhou, Dong-jie Feng, Yuan Fang, Feng-yan Zha, Er-hui Wang, Yan-zhen Li, Min-xin Wei, Jun-min Wen

**Affiliations:** 1 Graduate School of Tianjin Medical University, No. 22 Qixiangtai Road, Tianjin, 300070 China; 2 Department of Critical Care Medicine ICU, Fu Wai Hospital, Chinese Academy of Medical Sciences, Shenzhen, 518057 Guangdong China

**Keywords:** Acute type a aortic dissection (ATAAD);blood platelet count;cardiopulmonary bypass (CPB);heparin-induced thrombocytopenia;heparin;thrombosis

## Abstract

**Purpose:**

The present study aimed to explore the clinical characteristics of heparin-induced thrombocytopenia (HIT) after surgery for acute type A aortic dissection and perform a relevant prognostic analysis.

**Methods:**

After continuous observation and analysis of 204 patients who underwent acute type A aortic dissection, we found that blood platelets decreased significantly after surgery and that these patients can be suspected to suffer HIT based on relevant 4Ts scores. For these suspected HIT patients, a latex particle-enhanced immunoturbidimetric assay was conducted to detect heparin-induced antibodies. Perioperative clinical data of patients in HIT and non-HIT groups were recorded as were blood platelet counts, HIT antibody test results, 4Ts scores, thromboembolic complications, clinical prognosis and outcomes.

**Results:**

In the present study, 38 suspected HIT patients, 16 HIT patients and 188 non-HIT patients were selected in the clinical setting. Among them, HIT patients were found to have prolonged cardiopulmonary bypass time (223 min on average vs. 164 min) and delayed aortic cross-clamp time (128 min on average vs. 107 min), and these differences between HIT patients and non-HIT patients were significant (*P* < 0.05). Additionally, the HIT group required longer operation time and higher dose of heparin, but showing no statistical differences (*P* > 0.05). The transfusions of blood platelets in the HIT group and non-HIT group were 18.7 ± 5.0u and 15.6 ± 7.34 u, respectively. In the HIT group, the mechanic ventilation time and the length of ICU stay were longer comparing the non-HIT group(*P* < 0.05), though no significant differences in total length of stay or In-hospital mortality were observed (*P* > 0.05). The incidence of continuous renal replacement therapy in HIT group was higher than the non-HIT group (*P* < 0.05). Additionally,there were no significant differences in 24-h postoperative drainage or reoperation for bleeding in both group(*P* > 0.05). However, the HIT antibody titer in the HIT group was significantly higher than that in the Suspected HIT group (2.7 ± 0.8 U/mL vs. 0.3 ± 0.2 U/mL) (*P* < 0.05). Among patients diagnosed with HIT, the incidence of thromboembolism reached 31.5%.For example, two HIT patients newly developed thromboembolism in both lower extremities,and three patients experienced cerebral infarction.

**Conclusions:**

After surgery for acute type A aortic dissection, HIT patients developed postoperative complications, the duration of ventilatory support and length of ICU stay were extended, and the incidence of thromboembolism increased. HIT antibody detection and risk classification should be implemented for high-risk patients showing early clinical characteristics.

## Introduction

Heparin-induced thrombocytopenia (HIT) is an iatrogenic disease that potentially threatens the lives of people exposed to heparin or low-molecular-weight heparin. Because of the use of heparin during cardiopulmonary bypass, patients undergoing cardiac surgeries are at risk of HIT [1]. For example, risk of thrombosis mediated by heparin platelet factor 4 (PF-4) complex antibody in the circulation was found to be 30 times higher than that of the general population [2]. A reduction in blood platelet count (PLTc) after surgery for acute type A aortic dissection (ATAAD) commonly occurs in the clinical setting. However, HIT induced by heparin during cardiopulmonary bypass is rarely reported. Furthermore, no specificity in clinical manifestations of HIT has been found, which mainly include PLTc decrease at several days or months after heparin therapy along with the presence or absence of a newly developed thromboembolism. In this case, a contradictory state between a decrease in PLTc and thrombosis occurs. This poses a great challenge to clinical treatment [3]. In this study, patients undergoing acute type A aortic dissection were observed for signs of HIT, and clinical outcomes after surgery were assessed. Hopefully, appropriate support can be provided for clinical prevention and prognosis.

## Methods

### Study design and participants

The investigation was designed as a single-center,observational,prospective study. A total of 204 patients undergoing acute type A aortic dissection surgery were continuously observed from August 2018 to December 2019 in our medical center, Chinese Academy of Medical Sciences. ethics committee approval (Institutional Review Board File 201,810), signed the clinical research informed consent, The following inclusion criteria were applied:severe chest pain and acute type A aortic dissection confirmed by computed tomography, an onset time of fewer than 2 weeks, and patients scheduled for emergency operation. Exclusion criteria included the following:patients with congenital or acquired coagulation disease,chronic diseases of the liver or renal,recent use of anticoagulant or antiplatelet therapy within 5 to 7 days before surgery. We incorporated clinical assessment into the preliminary screening of HIT the using “4Ts” score (thrombocytopenia, timing of platelet count fall, thrombosis or other sequelae, and other causes for thrombocytopenia),The “4Ts” score are used to calculate the pretest clinical probability of HIT. All ATAAD patients were routinely evaluated by 4Ts scoring system, For patients undergoing cardiopulmonary bypass procedure who were admitted to our center during the same period,and patients undergoing endovascular repair of type B aortic dissection,if a PLTc decrease above 50% is suspected to have HIT in the clinic, (compared with its baseline value before surgery) after the administration of heparin during the cardiopulmonary bypass or endovascular repair,we obtained the 4Ts scores [4, 5]; if the score is ≥4 points,we would test an antiplatelet factor 4-heparin antibody (HIT antibody) before surgery,and continuing observing the changes of HIT antibody until the 10th day after the operation.

### Laboratory assays

HIT antibody detection: HIT-Ab (anti-PF4/H antibody) in human citrate plasma was examined by a fully automatic latex particle-enhanced immunoturbidimetric assays using an ACL TOP 300 (Werfen, USA). Regarding samples collected from patients undergoing heparin therapy, the results may be considered positive if the anti-PF4/H antibody detection kit shows that the corresponding value is no less than a threshold of 1.5 OD or U/mL (the same below). This signifies the presence of HIT Ab [6]. According to the algorithm model that can quantitate the probability of HIT [5],in our study, HIT was ruled out if the 4Ts score < 4 points and anti-PF4/H antibody < 0.6 OD. If the 4Ts score ≥ 6 points and anti-PF4/H antibody ≥1.8 OD,patient would be diagnosed as HIT. If the 4Ts score ≥ 4 points but anti-PF4/H antibody < 1.0 OD,the patient would be regarded as suspected HIT.

Collection of clinical data: ① The baseline information of all hospitalized patients with acute type A aortic dissection, including sex, age, body mass index, medical history (e.g.hypertension, diabetes, smoking or severe COPD), continuous renal replacement therapy (CRRT) or not, preoperative ejection fraction and PLTc, were obtained. ② Data on the time of extracorporeal circulation, time of aortic occlusion, time of circulation arrest, volume of blood platelets transfused during the surgery and heparin dosage were collected. ③ Complications during hospitalization and prognosis of all patients were also recorded.

### Surgical management

The procedure was performed under general anesthesia with tracheal intubation. The cardiopulmonary bypass procedure started after the systemic heparinization (heparin dosage 300 U/kg and maintaining ACT at more than 480 s). Major surgical procedures for these patients is to perform aortic arch replacement with a graft with four branches, and a particular stented endovascular graft is implanted in the true lumen of the descending thoracic aorta. When the artificial four-branch blood vessel is connected, a catheter can be intubated to restore the perfusion of the lower half of the body. Two branches of the artificial endograft are anastomosed with the left common carotid artery and the left subclavian artery. After the blood oxygen saturation rises above 85%, rewarming is started, and the proximal quadrant artery is then anastomosed. After completing the graft anastomosis for the two major aortic vessels, the ascending aorta is then incised to complete the procedure by anastomosing the innominate artery with the fourth endograft branch vessel.

### Statistical analysis

Statistical analysis was conducted using SPSS 19.0 software. Continuous variables with normal distribution are expressed as x ± s; variables with nonnormal distribution are expressed as medians and the 25% or 75% fractile. Categorical variables are expressed as percentages. To compare baseline information, an unpaired t-test and a chi-square test were conducted. Mann-Whitney U-test was used between the HIT group and the non-HIT group that data did not exhibit a normal distribution. Statistically significant differences were considered at a *P* value < 0.05.

## Results

A total of 204 patients with acute type A aortic dissection were consecutively included and observed. They were grouped according to 4Ts scores and anti-PF4/H antibody, and Among the 38 patients with 4Ts score ≥ 4 points, and anti-PF4/H antibody < 0.4 OD, 22 patients were classified as the suspected HIT group. Sixteen patients were eventually confirmed as HIT because 4Ts score ≥ 6 points, and anti-PF4/H antibody ≥1.8 OD [5]. Specifically, 16 and 188 patients were assigned to the HIT group and the non-HIT group respectively, respectively. The results of the comparison between the two groups are presented in Tables 1, 2 and 3. Through comparison of preoperative data, no significant differences were observed in age, sex or body mass index between the HIT and non-HIT groups (*P* > 0.05). Moreover, the HIT group was not significantly different from the non-HIT group in terms of the incidence of hypertension, diabetes, history of smoking, severe COPD or ejection fraction (*P* > 0.05). Moreover, patients in the HIT group had prolonged cardiopulmonary bypass time (223 min on average vs.164 min, *P* < 0.01) and a long aortic cross-clamping period (128 min on average vs.107 min, *P* < 0.05), with significance (*P* < 0.05). Although prolonged cardiopulmonary bypass time and a comparatively higher dose of heparin were observed in the HIT group, no significant difference from the dose of heparin in the non-HIT group was observed (*P* > 0.05). Furthermore, no significant differences were found in the volumes of blood platelets transfused between the two groups (18.7 ± 5.0 u vs. 15.6 ± 7.34 u). The mechanical ventilation time and length of ICU stay were longer in the HIT group, which was significantly different from those in the non-HIT group (*P* < 0.05). However, no significant differences in the total length of stay or the all-cause mortality were found between the two groups. Stroke incidence and CRRT occurrence rates in the HIT group were higher than those in the non-HIT group (*P* < 0.05). Twenty-four hours after the surgery, no significant differences were found in the drainage fluid amounts or second thoracotomy. Moreover, the HIT antibody titer in the HIT group (2.7 ± 0.8 U/mL) was much greater than that in the suspected HIT group (0.3 ± 0.2 U/mL) (Table 4, *P* < 0.05). In addition, the HIT and suspected HIT groups presented no significant differences in terms of 4Ts scores and minimum PLT and PLT decrease. Regarding the 16 patients in the HIT group, heparin or low-molecular-weight heparin treatment was immediately discontinued when HIT was suspected during evaluation in the clinic. Moreover, the agents used for monitoring arterial tube washing in 38 patients with suspected cases were replaced with argatroban.

**Table 1:** Preoperative data comparison between HIT and non-HIT groups

Parameters	All patients (*n* = 204)	HIT group (*n* = 16)	Non-HIT group (*n* = 188)	*P* value
Age (years)	51.0 ± 13.1	52.0 ± 13.3	51.5 ± 13.0	0.879
Sex (M/F)	168/36	10/6	158/30	0.737
BMI (kg/m^2^)	24.8 ± 3.8	25.5 ± 2.5	24.8 ± 3.9	0.112
Hypertension,n (%)	141 (69.1)	11 (68.8)	130 (69.1)	0.816
DM,n (%)	22 (10.7)	2 (12.5)	20 (10.6)	0.560
History of smoking	82 (40.2)	5 (31.3)	77 (40.9)	0.343
Renal replacement therapy,n (%)	0 (0)	0 (0)	0 (0)	1.000
Preoperative platelet count(× 10^9^/L)	195.8 ± 79.8	212.9 ± 67.3	192.8 ± 82.5	0.211
COPD,n (%)	3 (1.5)	0 (0)	3 (1.6)	0.584
LVEF (%)	57.8 ± 7.8	60.2 ± 3.2	57.7 ± 7.7	0.201

*BMI* body mass index, *DM* diabetes mellitus, *COPD* chronic obstructive pulmonary disease, *LVEF* left ventricular ejection fraction

**Table 2:** Intraoperative data comparison between the HIT group and the non-HIT group

Parameters	All patients (*n* = 204)	HIT group (*n* = 16)	Non-HIT group (*n* = 188)	*P* value
Renal replacement therapy,n (%)	16 (7.8)	7 (43.8)	9 (4.8)	0.001
In-hospital mortality,n (%)	10 (4.9)	2 (12.5)	8 (4.3)	0.622
Postoperative stroke,n (%)	10 (4.9)	4 (25)	6 (3.2)	0.001
Mechanic ventilation time (hours)	42.7 ± 53.2	93.2 ± 83.9	36.1 ± 47.7	0.017
Length of ICU stay (hours)	142.7 ± 123.2	226.5 ± 136.9	126.6 ± 110.9	0.012
24-h postoperative drainage (mL)	537.0 ± 262.5	536.9 ± 222.1	537.1 ± 384.7	0.997
Length of in-hospital (days)	27.7 ± 12.9	22.5 ± 10.6	28.0 ± 12.4	0.084
Reoperation for bleeding,n(%)	14 (6.8)	3 (18.7)	11 (5.8)	0.095

**Table 3:** Postoperative data comparison between HIT and non-HIT groups

Parameters	All patients (*n* = 204)	HIT group (*n* = 16)	Non-HIT group (*n* = 188)	*P* value
CPB time (min)	175.8 ± 63.6	223.1 ± 11.4	164.9 ± 59.8	0.006
Cross-clamp time (min)	111.8 ± 42.4	128.9 ± 30.1	107.1 ± 45.5	0.042
DHCA time (min)	17.9 ± 7.0	20.6 ± 7.1	16.6 ± 7.1	0.085
Intraoperative platelet (unit)	16.0 ± 6.6	18.7 ± 5.0	15.6 ± 7.3	0.131
Heparin dose (mg)	336.1 ± 80.2	407.1 ± 192.9	325.9 ± 77.2	0.081

*CPB* cardiopulmonary bypass, *DHCA* deep hypothermia circulatory arrest

**Table 4:** Comparison of HIT and suspected HIT groups

Parameters	All patients (*n* = 38)	HIT group (*n* = 16)	Suspected HIT group (*n* = 22)	*P* value
4Ts score	5.4 ± 1.2	5.7 ± 1.2	5.3 ± 1.1	0.527
preoperative HIT Ab (OD)	0.4 ± 0.2	0.5 ± 0.1	0.4 ± 0.1	0.651
HIT Ab titer (OD)	1.4 ± 1.6	2.7 ± 0.8	0.3 ± 0.2	0.001
PLT drop(%, 95 CI)	70.1 (67.9 ~ 79.0)	73.6 (67.2 ~ 79.4)	67.9 (62.9 ~ 71.7)	0.236
Minimum PLT(10^9^/L)	44.4 ± 16.6	40.9 ± 17.2	47.6 ± 20.1	0.076

## Discussion

The above mentioned data were collected from patients who underwent surgery in the aorta. In our study,the incidence of HIT was 7.8%(16/204) for specific patients who underwent surgery under deep hypothermic circulatory arrest (DHCA), it’s really surprising. But for the patients undergoing cardiopulmonary bypass with heparin during the same period,the incidence of HIT was 1.2%(16/1364). Regarding thoracotomy, cardiopulmonary bypass is associated with the anticoagulation process of heparin. For this reason, patients with acute type A aortic dissection undergo a longer procedure and a short DHCA process during surgery. All patients in this series were exposed to a large dose of heparin, and postoperative PLTc reduction for unknown causes increases the risk for HIT development. HIT onset is not easy to perceive due to a lack of specificity in clinical manifestations, though a PLTc reduction 3 days after heparin administration is the most common. Occasionally, such a manifestation may be observed within 24 h or several months after the application of heparin [7].

The findings of the present study strengthen the awareness that HIT is a important complication worth causing our great attention after surgery for acute type A aortic dissection. Although the differences in preoperative data between the HIT group and the non-HIT group showed no statistical significance. Because of apparently longer extracorporeal circulation time and aortic cross-clamp time in the HIT group, the dose of heparin needs to be increased. In our study, the doses of heparin in both groups were not significantly different from each other (*P* > 0.05). The extension of the time of extracorporeal circulation may enable the body to be exposed to heparin for a longer time. Hence, achieving an appropriate ratio between heparin and PF-4 and produce pathogenic HIT antibodies becomes much more likely [8]. In this context, the time of extracorporeal circulation and aortic cross-clamp should be reduced to the greatest extent during surgery for acute type A aortic dissection. Thus, both the exposure dose of heparin and the time of exposure can be decreased. PLTc reduction induced by HIT after surgery for acute type A aortic dissection makes it more possible to administer renal replacement therapy after acute kidney injury. The pathogenesis might involve the influence of microvascular thrombosis development because microvascular thrombosis has the potential to reduce the volume of blood flow in the kidney, further lowering the glomerular filtration rate and finally leading to acute kidney injury [9]. Although HIT increases the rate of stroke incidence, both thromboembolism and microvascular blood flow disorder are the primary reasons why the risk of stroke increases in this group. Nonetheless, further investigations are warranted to identify the effects of PLTc reduction on AKI and stroke after cardiopulmonary bypass. Considering that a longer extracorporeal circulation time is also a critical cause of an increase in postoperative complications, corresponding causal relationships or interactions should be further explored and clarified as well. Both the duration of mechanic ventilation and length of ICU stay were apparently longer in the HIT group. Although the differences in the length of in-hospital were eventually found to be insignificant between the HIT and non-HIT groups and no statistical differences in in-hospital mortality rates were observed, the relevant treatment costs and risks increased. In contrast, HIT reduces the PLTc, but the 24 h postoperative volume of drainage fluid has not significant increase,and the probability of reoperation for bleeding does not increase at the same time, which may be related to the characteristics of HIT,that there are contradictions between PLTc reduction and thrombosis),conversely, the risk of hemorrhage does not increase.

HIT occurs along with arterial and venous thrombotic events; newly developed thromboembolism is the most serious clinical outcome of HIT, primarily including thrombosis in the lower limbs, pulmonary embolism, myocardial infarction, cerebral infarction, local necrosis in the heparin-injected site, gangrene, necrosis and hemorrhage in the adrenal gland [10]. Among the patients included in the present study, two patients had new-onset thromboembolism in both lower limbs, and three patients had postoperative cerebral infarction. The thromboembolism incidence rate reached 31.5%, greatly similar to the ratios (30–50%) reported in other countries [11]. If the PLTc decreases by over 50% or if thrombosis occurs, then suspected HIT is confirmed [12]. The 4Ts scoring system combines the following four characteristics: (1) amplitude of PLTc reduction, (2) duration of PLTc reduction after exposure to heparin, (3) presence of thrombosis,(4) absence of PLTc reduction due to other causes [13]. Such a system has been widely applied in the clinical setting. A score below four points indicates a high negative predictive value (97–100%), whereas that above six points suggests that the corresponding positive predictive value is low (40–82%) [14]. Clearly, the 4Ts scoring system has high sensitivity but low specificity, though it can be used as a preliminary screening method for exclusion of HIT in the clinical setting.

PLTc reduction is very common after surgery for acute type A aortic dissection, and true and false lumens are formed in the aorta after the onset of aortic dissection. Consequently, the blood flows through the intimal tear into the false lumen and thus comes into contact with the subendothelial tissues. In this way, coagulation and fibrinolysis systems are activated, leading to platelet activation and aggregation as well as the formation of blood clots [15]. In addition to extensive thrombosis in the false lumen, microthrombogenesis occurs in the whole body, causing the consumption of massive blood platelets and other blood coagulation factors. Therefore, both PLTc reduction and extensive thrombosis in the false lumen are closely related to platelet consumption [16]. The existing literature notes that expression of platelet-activating factor-4 is upregulated in the early stage of acute type A aortic dissection [17]. However, the correlation between platelet-activating factor-4 expression upregulation and HIT remains unclear. Regardless, there is no doubt that such upregulation increases the likelihood of binding after heparin exposure, which may be a reason for the occurrence of HIT. During extracorporeal circulation, the interaction of blood and the tube, together with the shear force of the loop, can lead to platelet activation. As a consequence, the blood constituents in peripheral circulation are consumed [18], though the platelet aggregation function may change after extracorporeal circulation. Accordingly, variations in surface marker expression, morphology and volume in blood platelets can be found [19]. Furthermore, PLTc reduction is associated with the administration of antiplatelet drugs, and 4Ts scores are therefore elevated. This may cause an increase in false-positive rates obtained through HIT diagnosis. Through 4Ts scoring, 38 patients in this study were suspected to have HIT, and these patients were divided into confirmed and suspected HIT groups based on laboratory testing of HIT antibodies. As shown in Table 4, the 4Ts scores of the confirmed HIT group showed no differences from those of the suspected HIT group. In terms of clinical manifestations, no significant differences in either absolute values of minimum PLT or PLT reduction were observed. Considering that the 4Ts scoring system has high sensitivity but low specificity [20], it should only be used as a preliminary screening method for preliminary exclusion of HIT in the clinical setting. Because of low specificity, HIT antibody detection needs to be further implemented to avoid overdiagnosis and incorrect treatment.

**Fig. 1:**
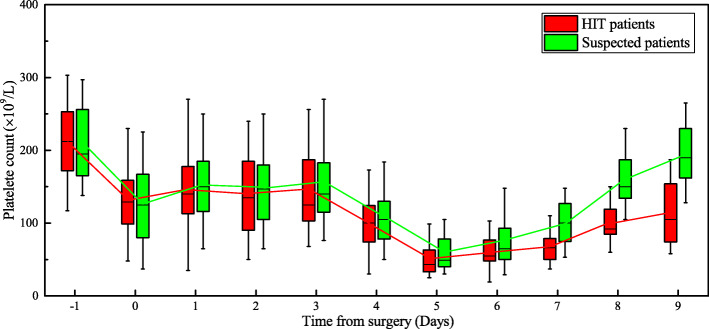
Comparison of platelet counts between HIT patients and Suspected HIT patients

**Fig. 2:**
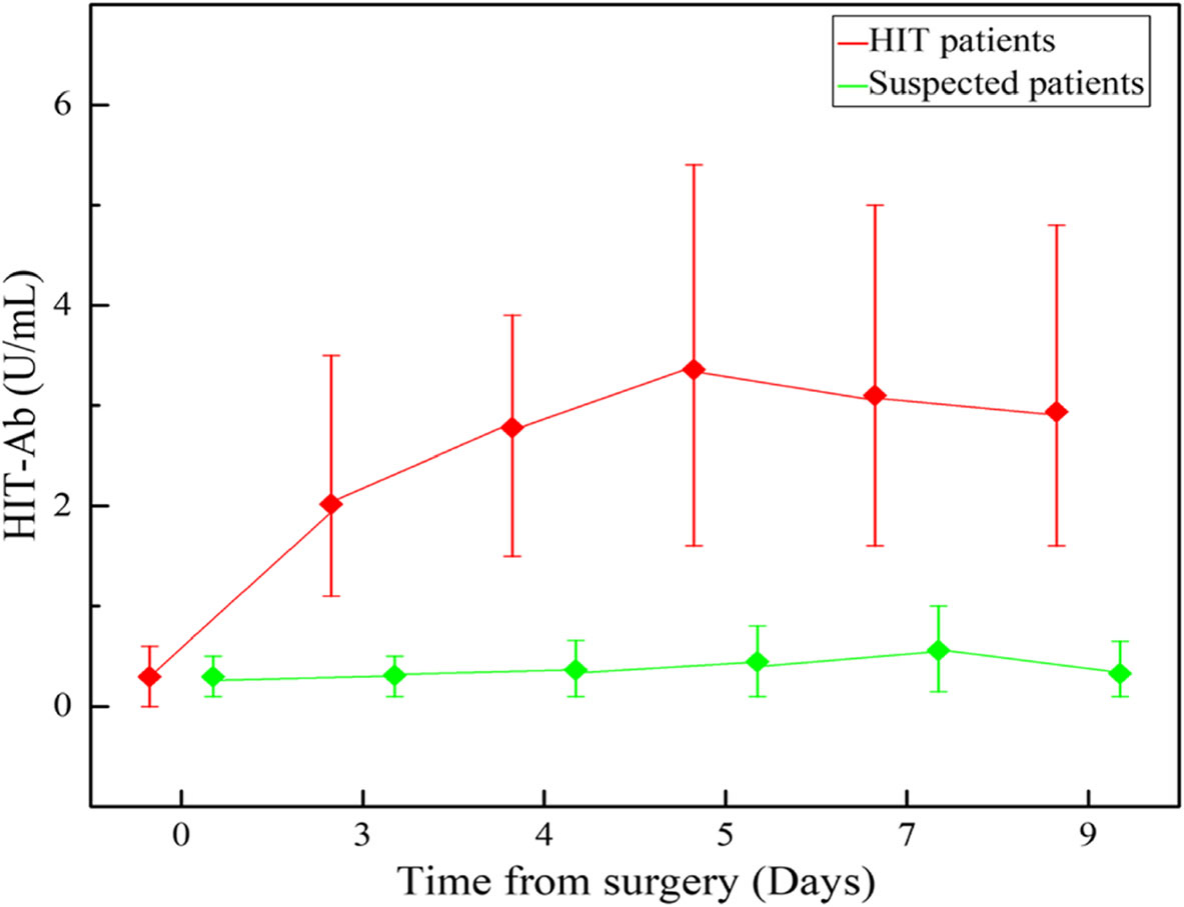
Comparison of HIT-Ab between HIT patients and Suspected HIT patients

As seen from the trend illustrated in Fig. 1 and Fig. 2, the HIT antibody concentration in the HIT group reached its peak on the fifth day after surgery,when the platelet count reduced at its lowest. Furthermore, the concentration of HIT antibodies in the confirmed HIT group (2.7 ± 0.8 U/mL) was significantly higher than that of the Suspected HIT group (0.3 ± 0.2 U/mL), providing a valid basis for early diagnosis of HIT in the clinical setting. Currently, the corresponding diagnostic measures principally include specific anti-HIT-IgG antibody and anti-HIT antibody complexes (e.g., IgG, IgA and IgM) [3]. Enzyme-linked immunosorbent assays, particle immune approaches and colloidal gold immunochromatography are commonly used manual testing methods. In our study, immunonephelometry was used to achieve rapid detection with a fully automatic coagulometer (15 min) [21]. IF choose the appropriate cutoff value, The negative predictive value obtained through anti-PF4/H antibody is nearly 99% [5, 22]. Therefore, HIT antibody detection can be utilized as a method of differential diagnosis targeted at patients suspected of having HIT. Moreover, as the variations in HTI antibody concentration have a negative correlation with PLTc increase, this measure can be adopted as an index for predicting an improvement in the patient’s response to clinical treatment. But for intermediate value of 4Ts score and anti-PF4/H antibody,for example 4Ts score ≥ 4 points, and anti-PF4/H antibody ≤1.5 OD,these threshold values will be weakly negative or weakly positive [5] . which may be particularly dangerous in patients with HIT. 5-hydroxytryptamine release from activated platelet function tests was recommended for all intermediate probabilities, although it is seldom applied because of methodological defects [23].

Undoubtedly, there are certain limitations in the present study. As single-center observational research, laboratory tests were not conducted to determine HIT function.

## Conclusions

Patients with HIT after surgery for acute type A aortic dissection have significantly increased postoperative complications and prolonged mechanical ventilation time and length of ICU stay, and the incidence of thromboembolism increased significantly. When there is an unexplained reduction in PLTc after surgery, it is necessary to be alert to the occurrence of HIT, especially when the time of cardiopulmonary bypass and aortic cross-clamping are long and the heparin dose is too large. HIT antibody testing and risk stratification should be performed for these early clinical high-risk patients.

## Data Availability

All data used in study are available available from the corresponding author on reasonable request.

## Abbreviations

ATAAD: Acute type A aortic dissection
ACC: Aortic cross-clamp
AKI: Acute kidney injury
BMI: Body mass index
CABG: Coronary artery bypass graft
COPD: Chronic obstructive pulmonary disease
CPB: Cardiopulmonary bypass
CRRT: Continuous renal replacement therapy
DHCA: Deep hypothermic circulatory arrest
DM: Diabetes mellitus
ELISA: Enzyme linked immunosorbent assay
HIT: Heparin-induced thrombocytopenia
ICU: Intensive care unit
LVEF: Left ventricular ejection fraction
